# The terpene synthase gene family in maize – a clarification of existing community nomenclature

**DOI:** 10.1186/s12864-023-09856-7

**Published:** 2023-12-06

**Authors:** Tobias G. Köllner, Jonathan Gershenzon, Reuben J. Peters, Philipp Zerbe, Eric A. Schmelz

**Affiliations:** 1 Department of Natural Product Biosynthesis, Max Planck Institute for Chemical Ecology, D-07745 Jena, Germany; 2 Department of Biochemistry, Max Planck Institute for Chemical Ecology, D-07745 Jena, Germany; 3 Roy J. Carver Department of Biochemistry, Biophysics & Molecular Biology, Iowa State University, Ames, IA 50011 USA; 4 Department of Plant Biology, University of California-Davis, Davis, CA 95616 USA; 5 Section of Cell and Developmental Biology, University of California, San Diego, La Jolla, CA 92093-0380 USA

## Main text

Terpenes are important natural products functioning in both the primary and specialized metabolism of plants, bacteria, fungi, and other life forms. Core structural diversity is mainly determined by terpene synthases (TPS), enzymes that convert ubiquitous prenyl diphosphates such as geranyl diphosphate, farnesyl diphosphate, and geranylgeranyl diphosphate into the various terpene backbones.

Terpene synthases in the crop plant maize (*Zea mays*) have been the subject of active research since the 1990s. A majority of maize TPS enzymes have already been functionally described and characterized, many of them in the laboratories of the authors of this clarification effort (reviewed in [1, 2]; Table 1). A comprehensive analysis of the *TPS* genes in the maize inbred lines B73 and W22 showed that both contain about 40 *TPS* genes, although the number varies between the different lines (43 in B73 versus 38 in W22) [3, 4]. These numbers include apparent pseudogenes, as it has been shown that a pseudogene in one maize line can be functional in another line [3, 5, 6].

**Table 1:** Current existing nomenclature of the terpene synthase gene family in maize. The first column contains the names already assigned to functionally characterized maize *TPS* genes in the literature (for citations see last column). To fill the remaining gaps in the *TPS* numbering and to unify the nomenclature, we propose the names listed in the second column, mainly following the original names or the names already given in Ding et al. [4] (third column). Please note that this table also includes obvious pseudogenes, because it has been shown that a pseudogene in one maize line can be functional in another line [3, 5, 6]

Name in the primary literature	Name proposed in this study	Ding et al. [4]	Sun et al. [7]	B73 reference GRAMENE 4.0	B73 reference NAM 5.0	Gene identifier (other maize lines)	Literature
*tps2*	*ZmTPS2*	*tps2*	*ZmTPS15*	Zm00001d015053	Zm00001eb230410		[9]
*tps3*	*ZmTPS3*	*tps3*		Zm00001d015054	Zm00001eb230440		[9]
*tps4*	*ZmTPS4*	*tps4*		Zm00001d024478	Zm00001eb415080		[5]
*tps5*	*ZmTPS5*	*tps5*		Zm00001d024481	Zm00001eb415090		[5]
*tps6/Zx1*	*ZmTPS6/Zx1*	*tps6*	*ZmTPS21*	Zm00001d024207	Zm00001eb412960		[10]
*tps7*	*ZmTPS7*	*tps7*	*ZmTPS4*	Zm00001d032230	Zm00001eb041770		[11]
*ZmTPS8*	*ZmTPS8*	*tps8*	*ZmTPS2*	Zm00001d029195	Zm00001eb017120		[12]
	*ZmTPS9*	*tps9*	*ZmTPS24*	Zm00001d024477	Zm00001eb415070		
*tps10*	*ZmTPS10*	*tps10*		Zm00001d024486	Zm00001eb415160		[13]
*tps11/Zx3*	*ZmTPS11/Zx3*	*tps11*		Zm00001d024210	Zm00001eb412960		[10]
*TPS12/Zx2*	*ZmTPS12/Zx2*	*tps12*		Zm00001d024208	Zm00001eb412960		[4]
*TPS13/Zx4*	*ZmTPS13/Zx4*	*tps13*		Zm00001d024211	Zm00001eb412990		[4]
	*ZmTPS14*		*ZmTPS9*	Zm00001d004484	Zm00001eb089110		
*tps15*	*ZmTPS15*	*tps15*	*ZmTPS16*	Zm00001d035682	Zm00001eb267020		[14]
	*ZmTPS16*		*ZmTPS26*	Zm00001d024667	Zm00001eb416710		
*ZmEDS*	*ZmTPS17/ZmEDS*	*tps17*	*ZmTPS10*	Zm00001d004509	Zm00001eb089360		[15]
	*ZmTPS18*	*tps18*	*ZmTPS8*	Zm00001d004279	Zm00001eb087570		
*tps19/stc1*	*ZmTPS19/STC1*	*tps19/stc1*	*ZmTPS19*	Zm00001d045054	Zm00001eb374210		[16]
*tps20*	*ZmTPS20*			Zm00001d024669	Zm00001eb416720		[14]
*tps21*	*ZmTPS21*	*tps21*	*ZmTPS20*	Zm00001d047440	Zm00001eb394330		[17]
*tps22*	*ZmTPS22*	*tps22*	*ZmTPS23*	Zm00001d024359	Zm00001eb414190		[14]
*tps23*	*ZmTPS23*	*tps23*	*ZmTPS22*	Zm00001d024234	Zm00001eb413120		[6]
	*ZmTPS24*		*ZmTPS14*	Zm00001d053916	Zm00001eb208380		
	*ZmTPS25*			Zm00001d053918	Zm00001eb208400		
*tps26*	*ZmTPS26*	*tps26*	*ZmTPS17*	Zm00001d037092	Zm00001eb278400		[16]
*ZmTPS27*	*ZmTPS27*	*tps30*	*ZmTPS1*	Zm00001d029139	Zm00001eb016730		[12]
	*ZmTPS28*		*ZmTPS18*	Zm00001d018611	Zm00001eb298110		
	*ZmTPS29*		*ZmTPS27*	Zm00001d029523	Zm00031ab020550		
	*ZmTPS30*			Zm00001d024479	Zm00001eb415090		
	*ZmTPS31*			Zm00001d024480	Zm00001eb415100		
	*ZmTPS32*		*ZmTPS29*			Zm00038ab090090	
	*ZmTPS33*		*ZmTPS30*			Zm00038ab090300	
	*ZmTPS34*		*ZmTPS31*			Zm00026ab417460	
	*ZmTPS35*		*ZmTPS28*			Zm00034ab064260	
	*ZmTPS36*			Zm00001d000337			
*an1/ZmCPS1*	*ZmTPS37/CPS1/AN1*	*an1/cps1*	*ZmTPS6*	Zm00001d032961	Zm00001eb048020		[18]
*an2/ZmCPS2*	*ZmTPS38/CPS2/AN2*	*an2/cps2*	*ZmTPS3*	Zm00001d029648	Zm00001eb021200		[19]
*ZmCPS3*	*ZmTPS39/CPS3*	*cps3*		Zm00001d024512	Zm00001eb415420		[20]
*ZmCPS4*	*ZmTPS40/CPS4*	*cps4*	*ZmTPS12*	Zm00001d048874	Zm00001eb167120		[20]
	*ZmTPS41/CPS5*			Zm00001d048867			
*ZmKSL1*	*ZmTPS42/KSL1*	*ksl1*	*ZmTPS13*	Zm00001d049957	Zm00001eb176190		
*ZmKSL2*	*ZmTPS43/KSL2*	*ksl2*	*ZmTPS11*	Zm00001d041082	Zm00001eb133200		[21]
*d5/ZmKSL3*	*ZmTPS44/KS(L3)/D5*	*ksl3*	*ZmTPS7*	Zm00001d002349	Zm00001eb071070		[22]
*ZmKSL4*	*ZmTPS45/KSL4*	*ksl4*	*ZmTPS5*	Zm00001d032858	Zm00001eb047160		[23]
*ZmKSL5*	*ZmTPS46/KSL5*	*ksl5*		Zm00001d002350	Zm00001eb071080		[22]
	*ZmTPS47/KSL6*	*ksl6*	*ZmTPS25*	Zm00001d024514	Zm00001eb415430		
*tps1*	*ZmTPS1/KSL7*	*tps1*		Zm00001d002351	Zm00001eb071090		[8, 22]

The amazing quantitative and qualitative plasticity of the maize *TPS* gene family was confirmed in a recent paper by Sun and coworkers, who analyzed *TPS* genes in the genomes of 26 inbred lines [7]. However, only 31 gene loci were included in this analysis, resulting in one-third of the already characterized *TPS* genes being omitted by the authors. Furthermore, the authors did not address the extensive pre-existing literature on maize terpene synthases prior to proposing a new nomenclature that was both incomplete and inconsistent with previously published names. Our concern with this approach is that it could lead to massive confusion in this field as readers will be unable to compare the new names with the original names without extensive sequence comparisons.

With the goal of minimizing confusion, we provide an overview of the existing maize nomenclature(s) and cite the primary literature in which the maize TPSs were first described and enzyme products characterized (Table 1). In addition, following the previously published *TPS* names, we propose to designate all mono- and sesquiterpene synthase genes with the abbreviation “*ZmTPS*” and a sequential numbering (*ZmTPS1* - *ZmTPS36*). Further we propose the continued designation of the class I diterpene synthase genes, namely kaurene synthase-like (KSL) genes, as *ZmTPS42/KSL1* to *ZmTPS47/KSL6*. Similarly, the class II diterpene synthase genes, namely the five copalyl diphosphate synthase (CPS) genes, are abbreviated as *ZmTPS37/CPS1* to *ZmTPS41/CPS5* (Table 1). Those involved in biosynthesis of the gibberellin hormone also have been designated by the original mutant names – i.e., an1/2 and d5, with the latter further modified as KS(L3)/D5 to highlight its activity as an *ent*-kaurene synthase. Note that this nomenclature includes not only the 43 *TPS* gene loci found in the B73 reference genomes GRAMENE 4.0 and NAM 5.0 (www.maizegdb.org), but also four additional *TPS* genes not present in B73 but identified in other maize lines by Sun and coworkers [7]. The improvement of the already sequenced genomes and the sequencing of additional maize lines will lead to continued changes in the absolute number of known maize *TPS* genes in the future. Therefore, the nomenclature proposed here is itself evolving and merits periodic revision that builds upon existing knowledge.

The overview of the maize *TPS* gene family presented in this paper, together with the proposed nomenclature that includes all previously published names, is intended to help to minimize confusion about maize *TPS* names. In addition, the list of uncharacterized *TPS* genes presented in Table 1 can serve as a reference point to motivate future research on TPSs and their biological roles in maize.

## Data Availability

Not applicable.

## Abbreviations

TPS: Terpene synthase
KSL: Kaurene synthase/kaurene synthase-like
CPS: Copalyl diphosphate synthase
